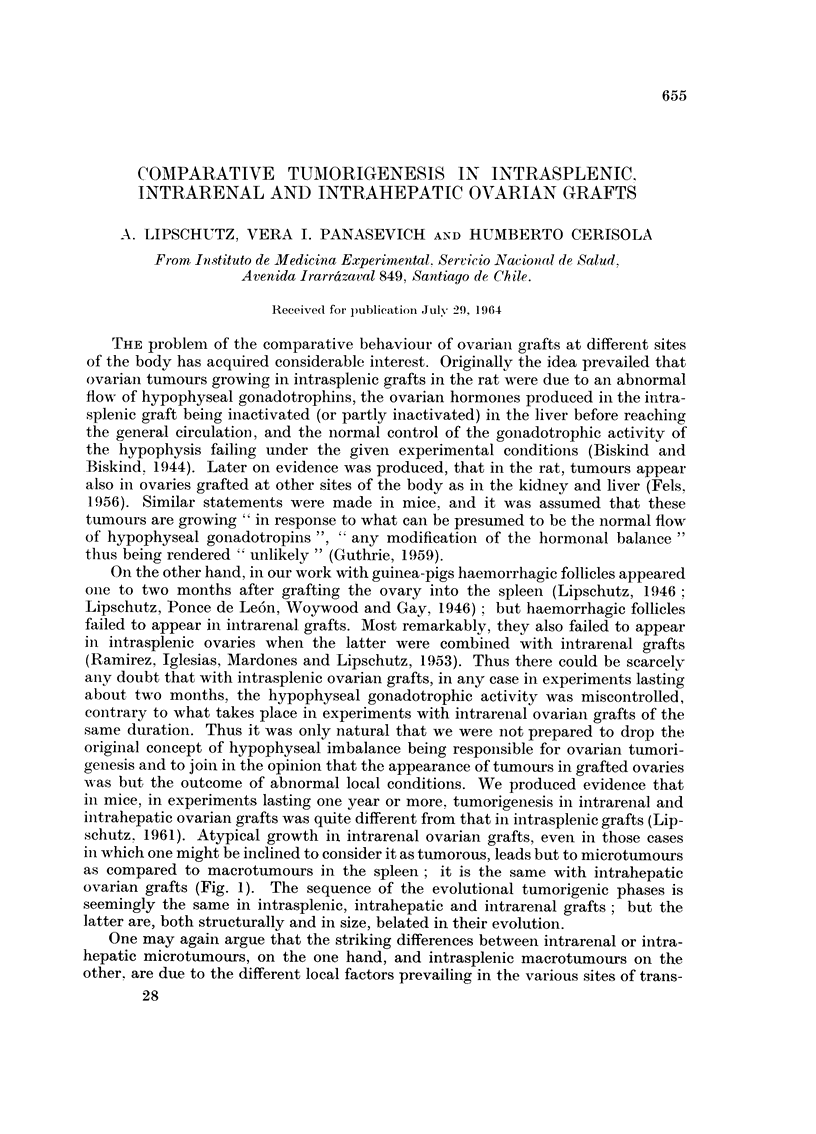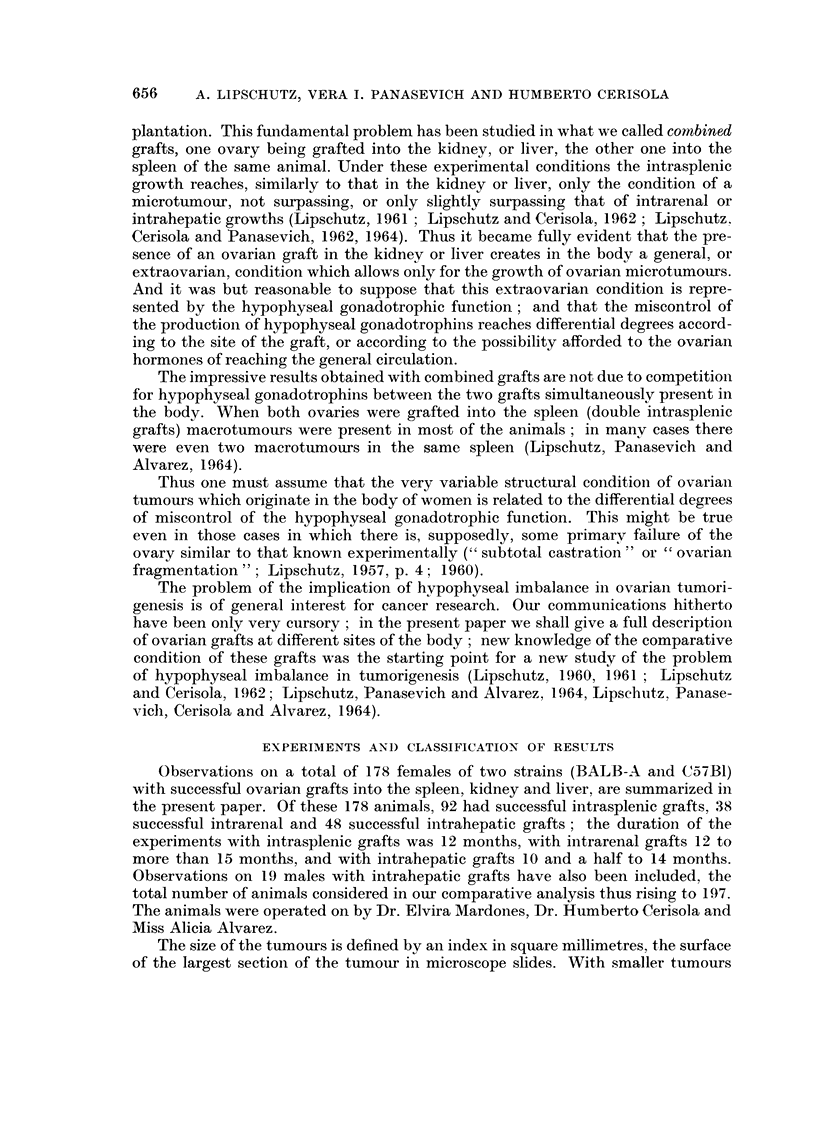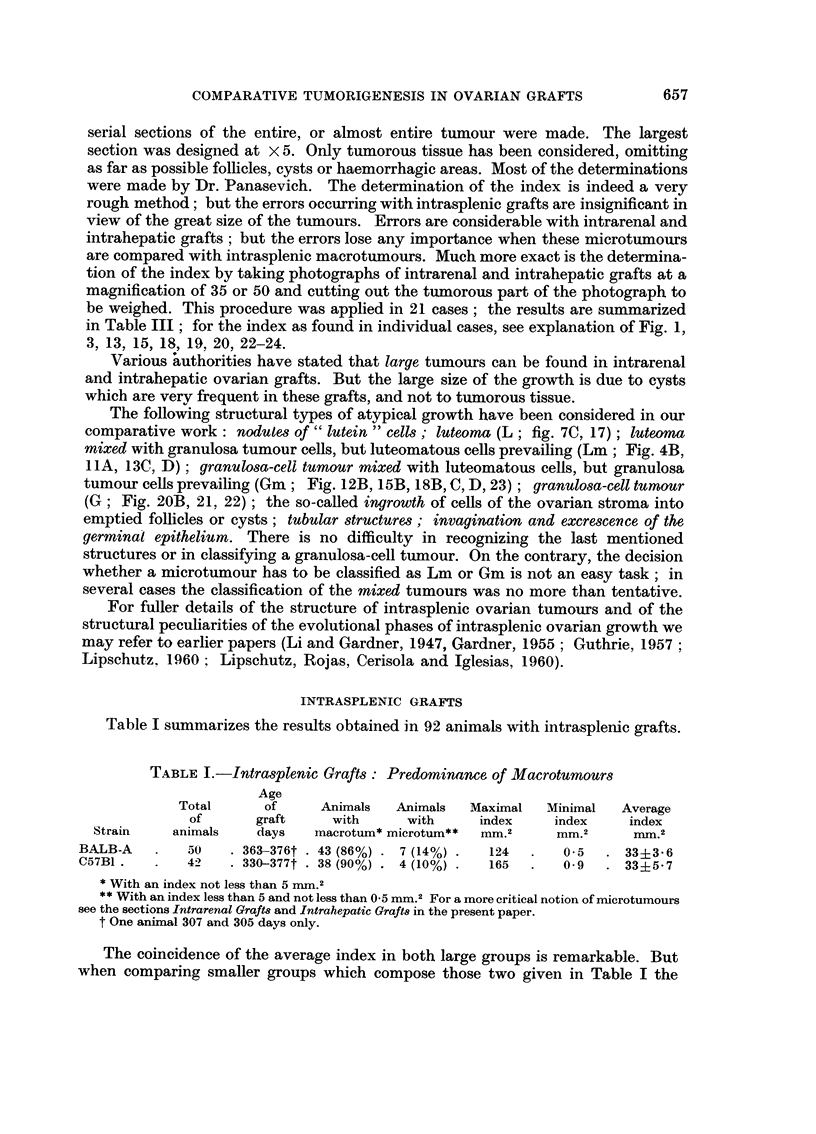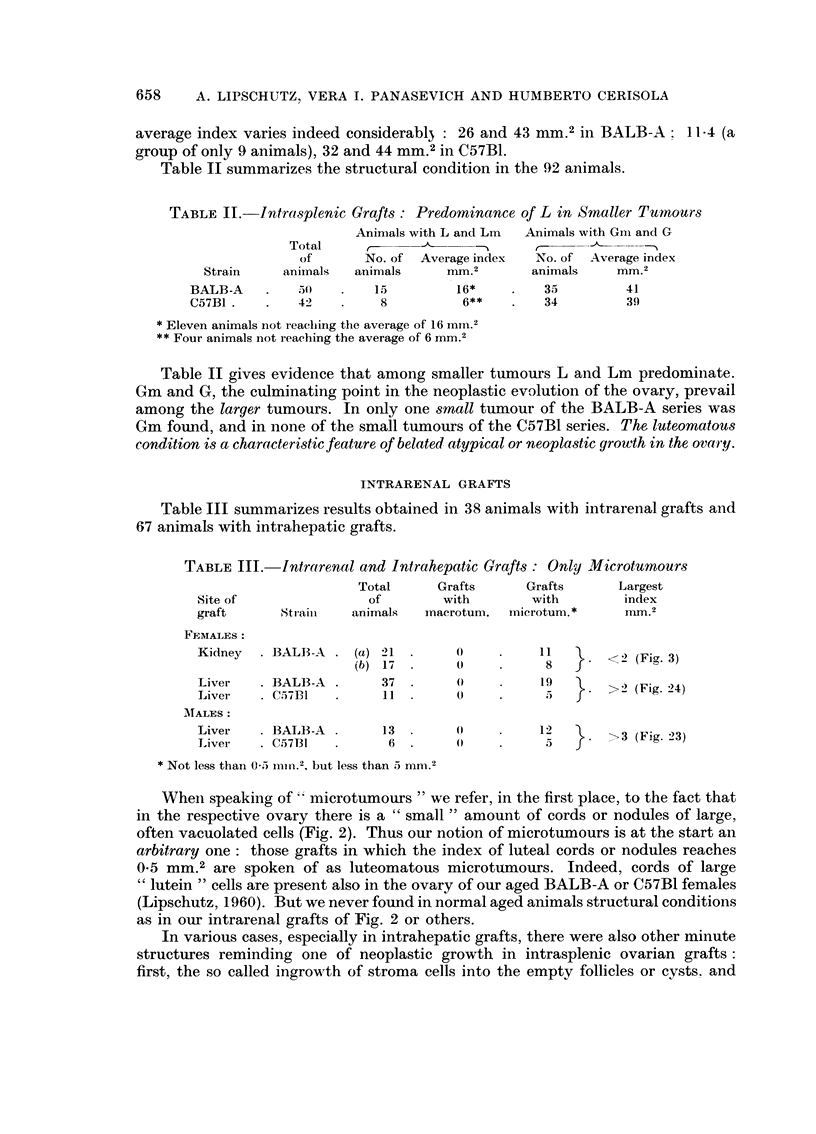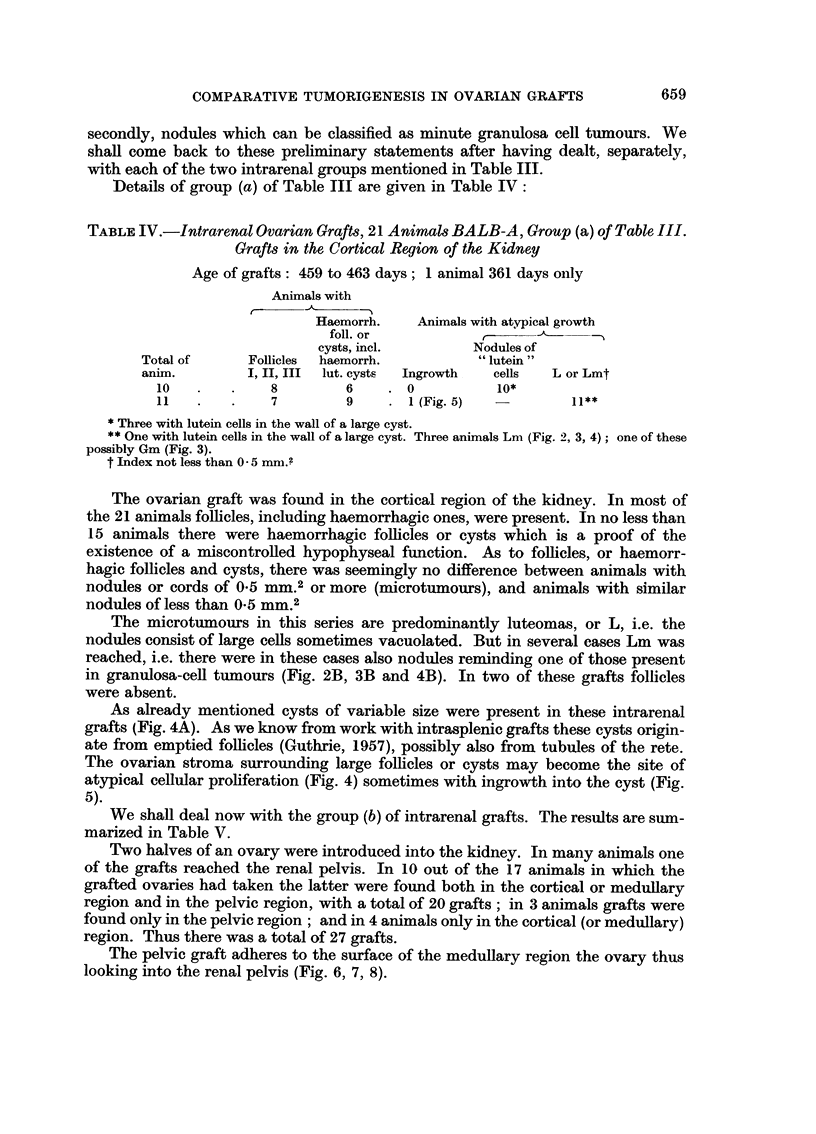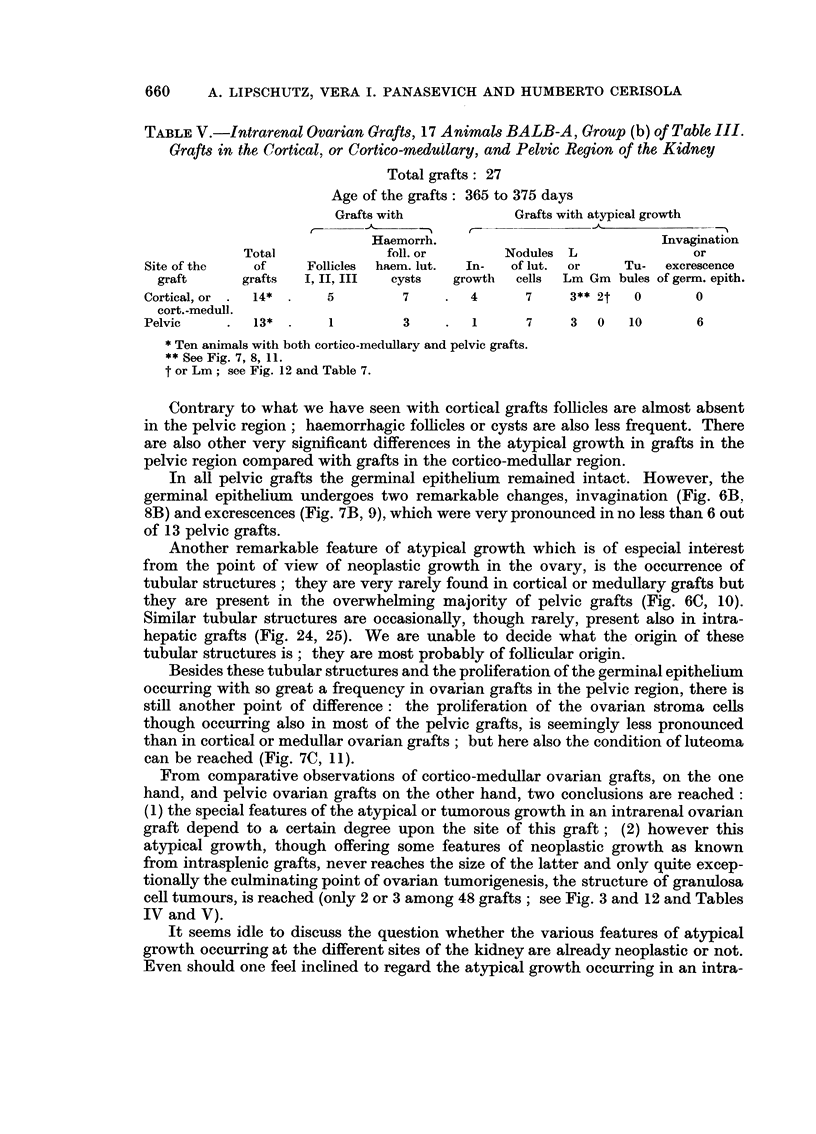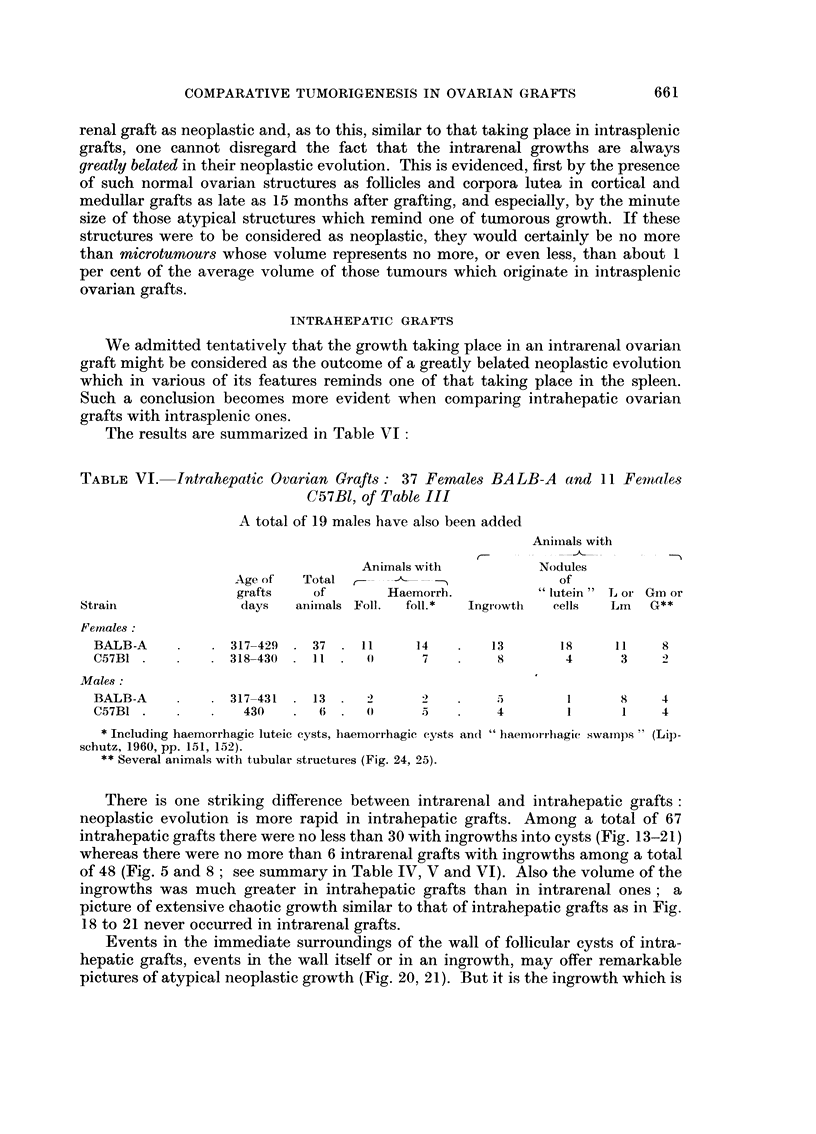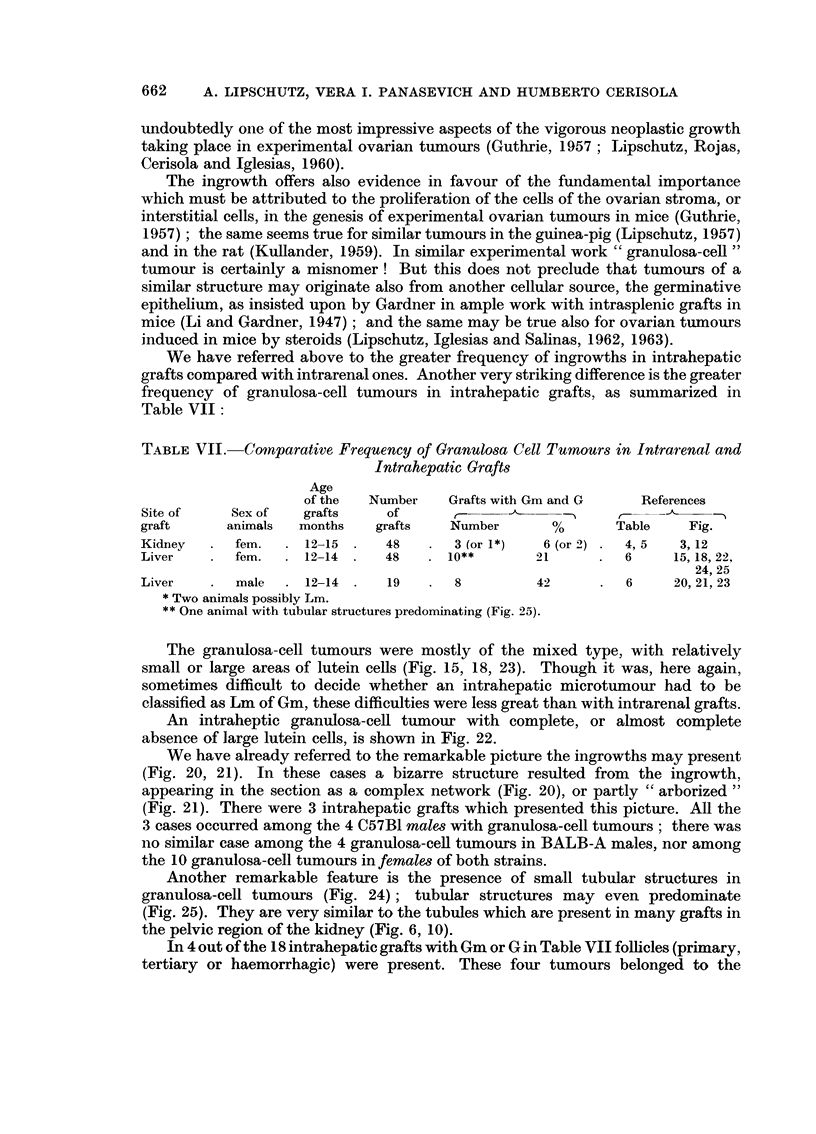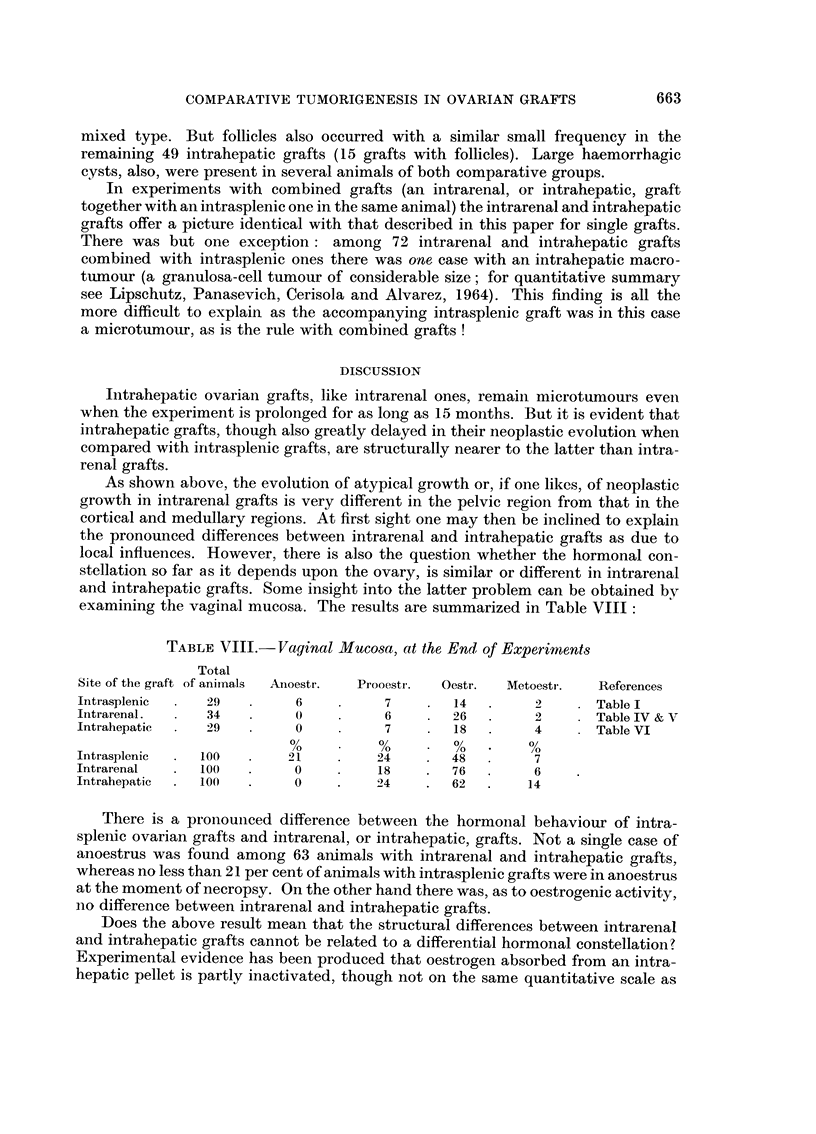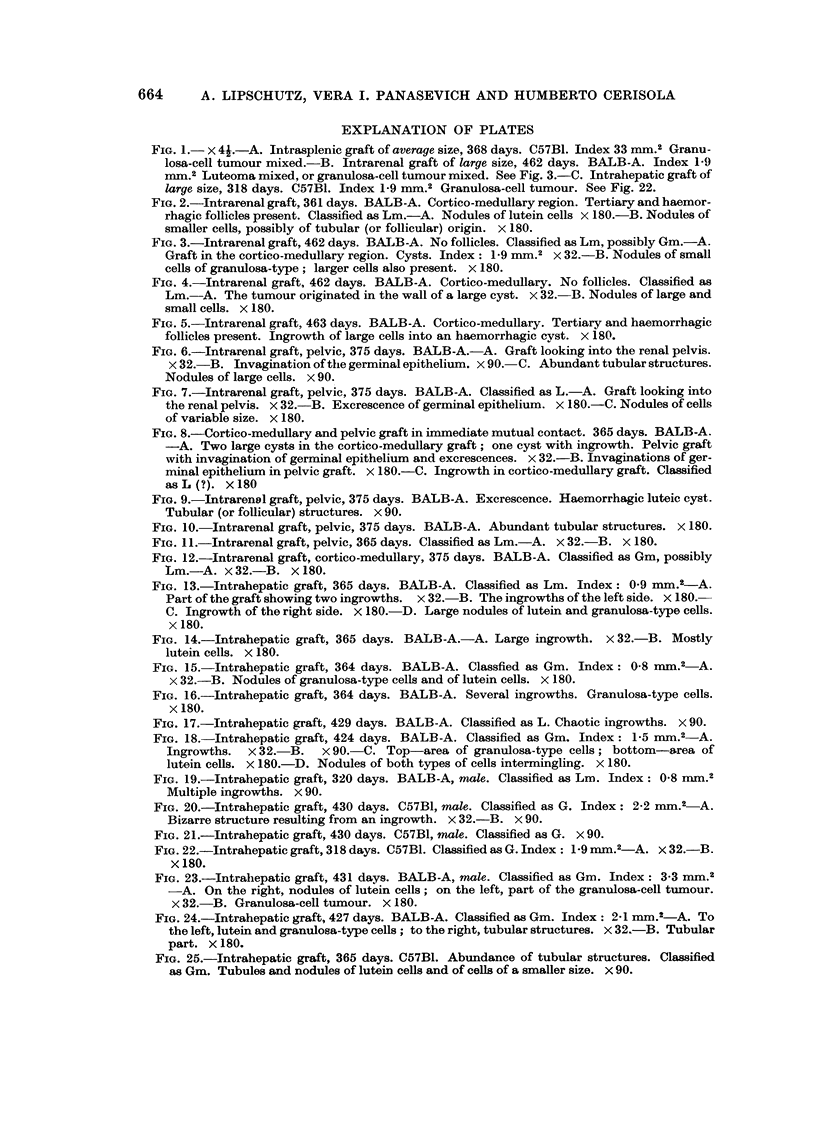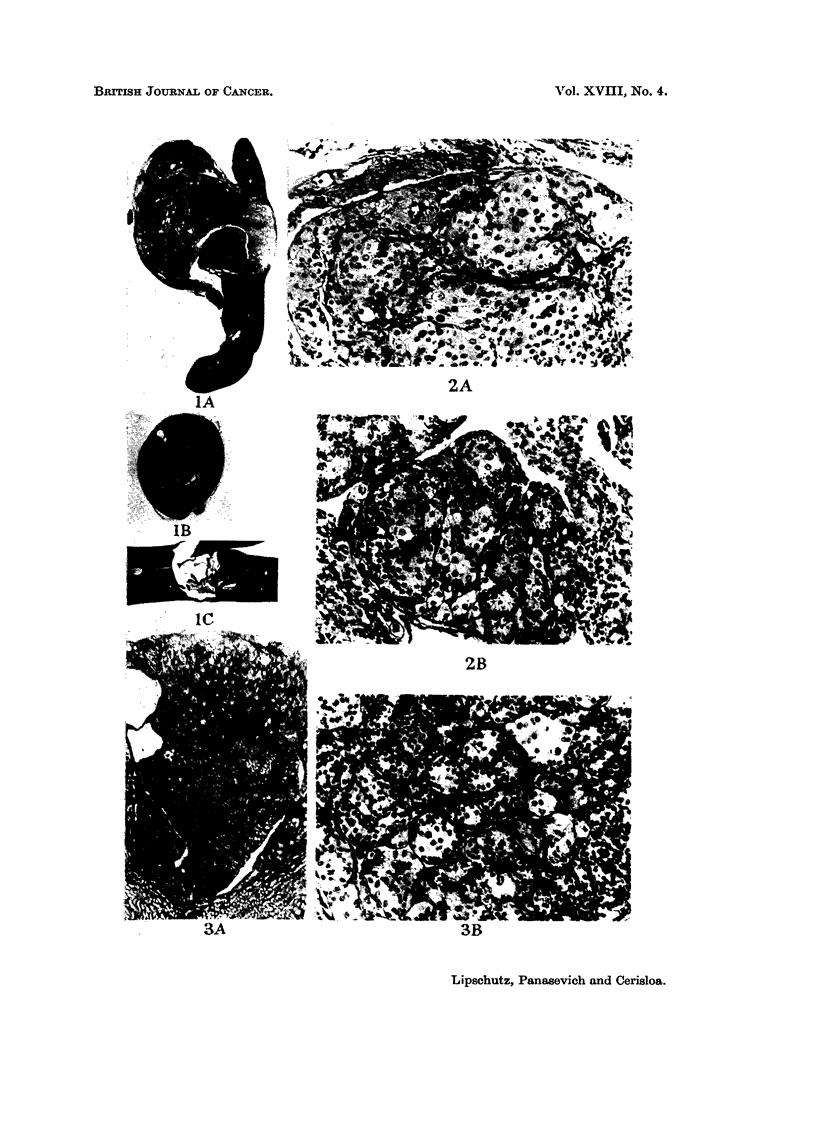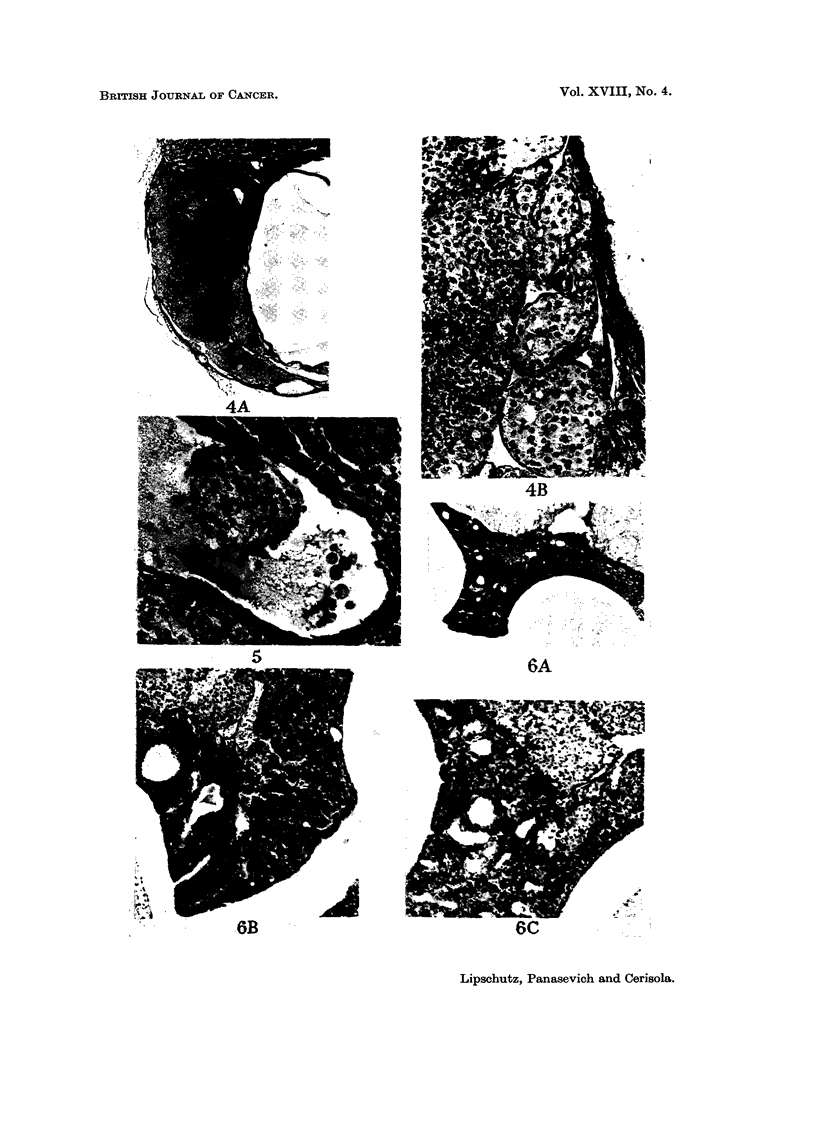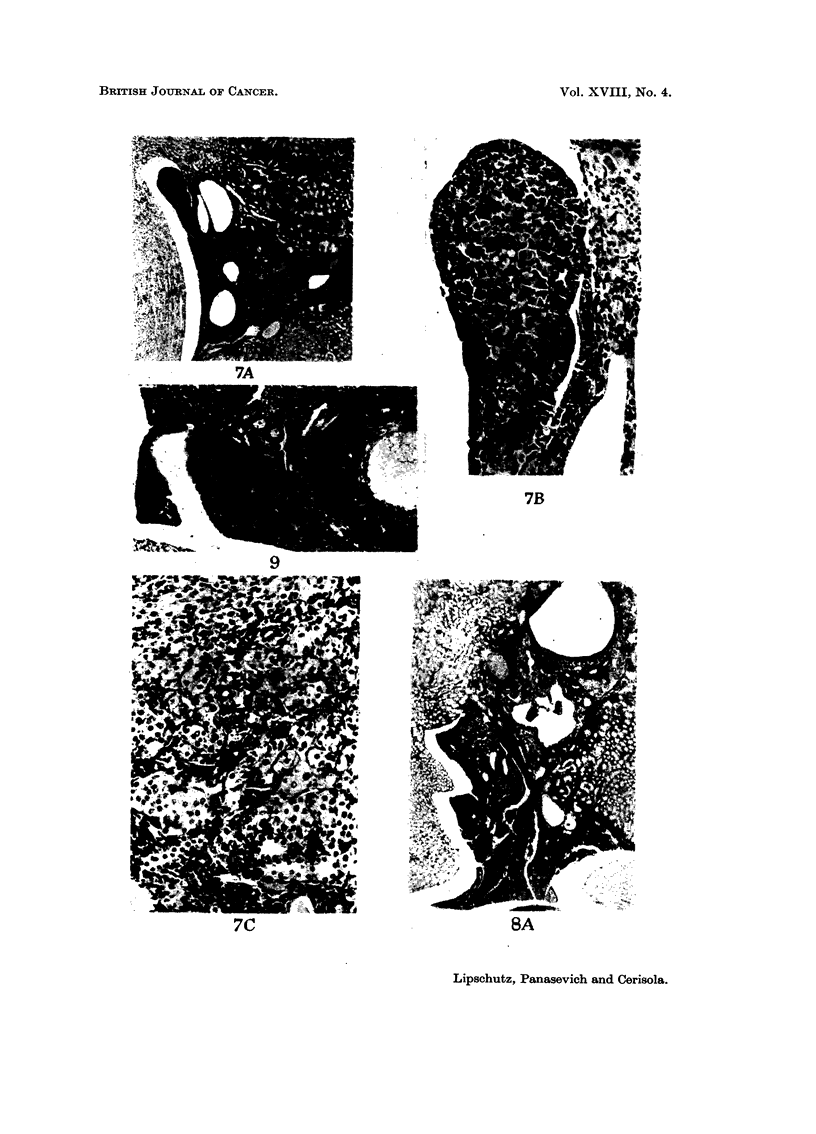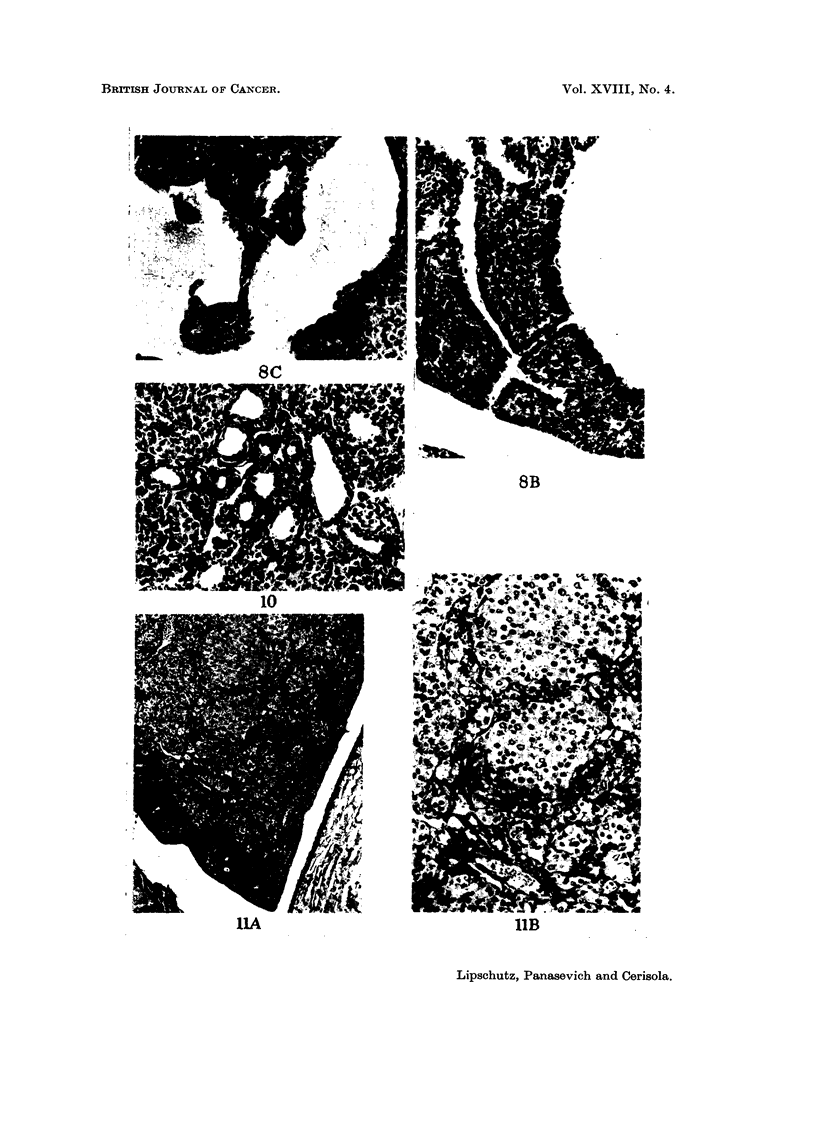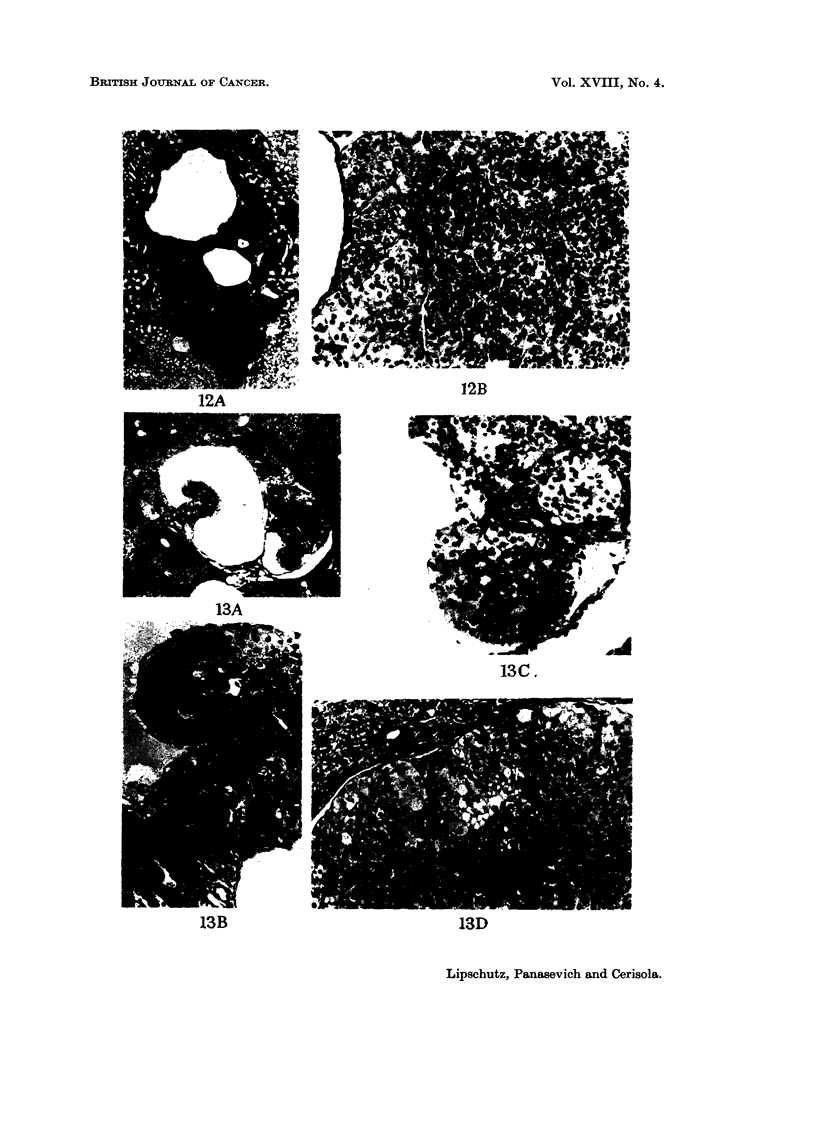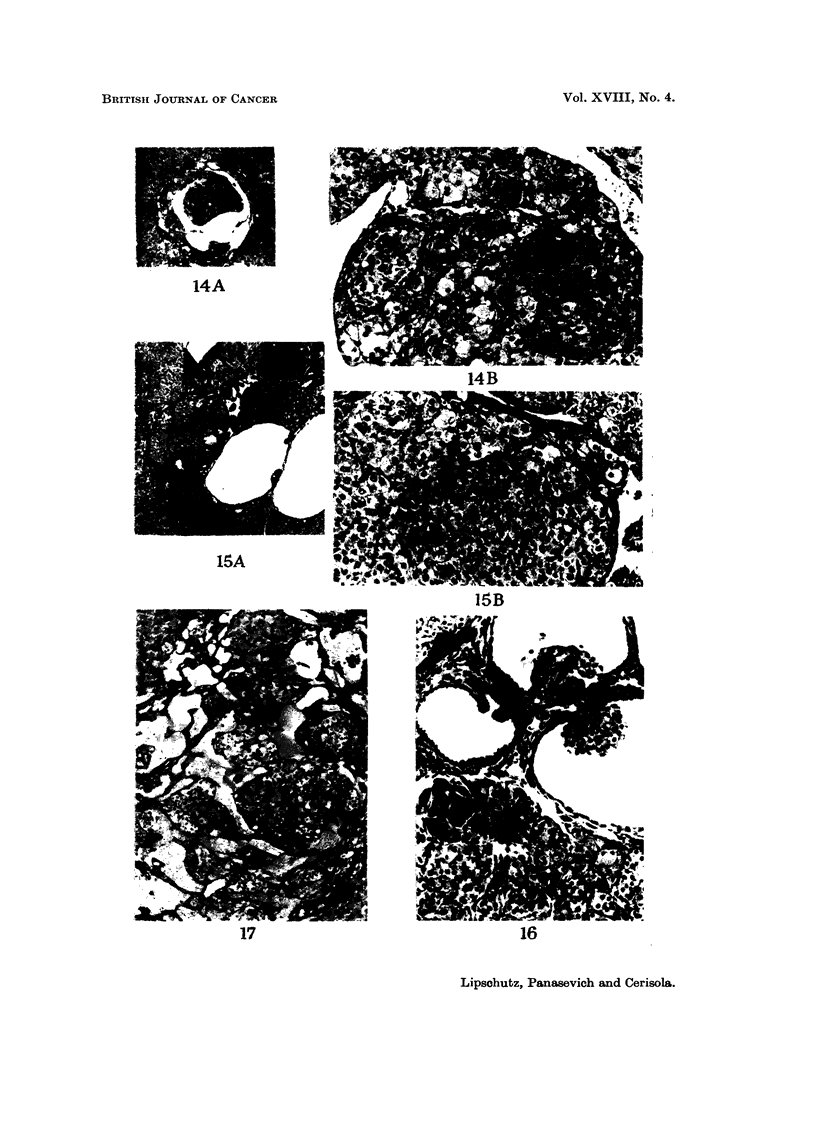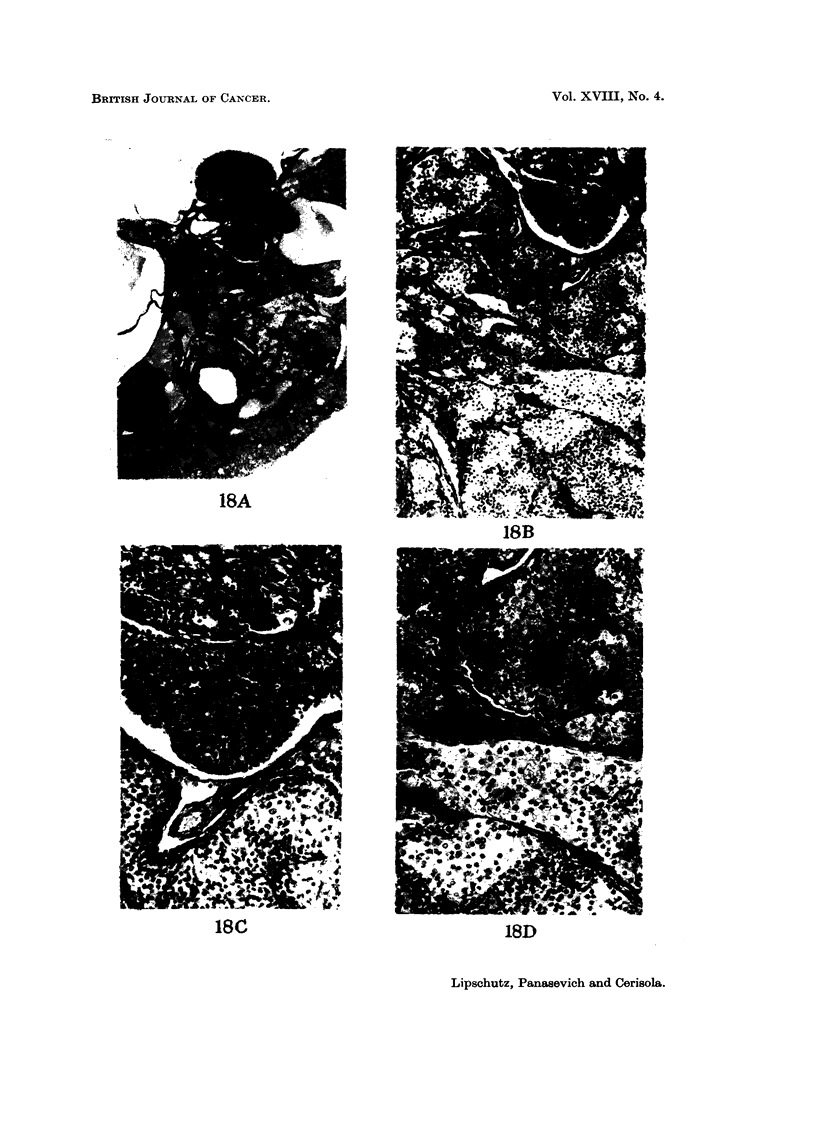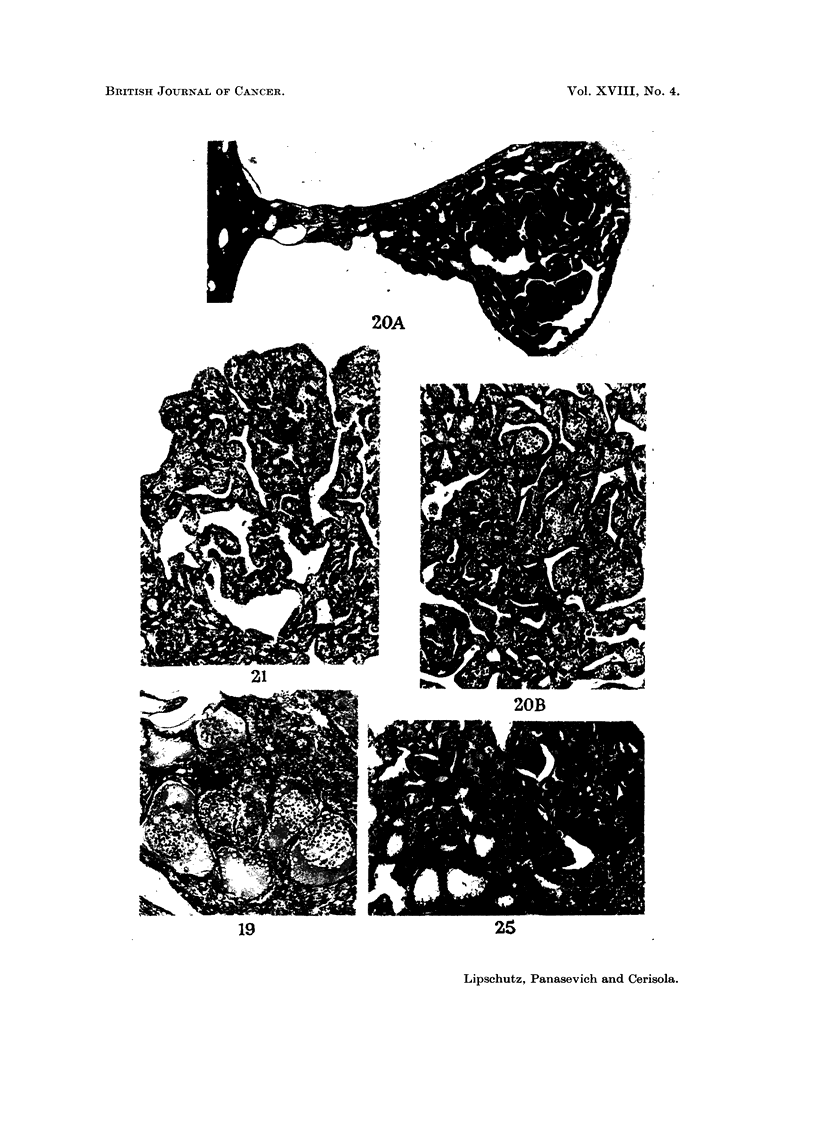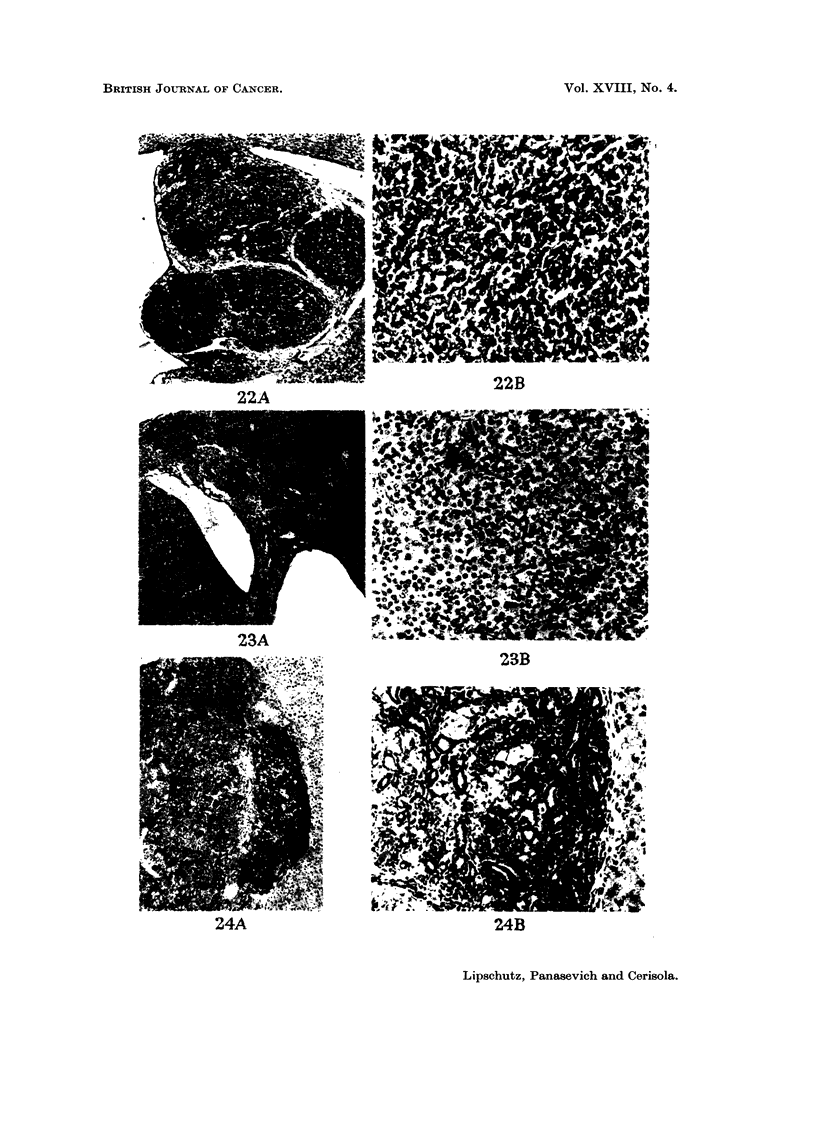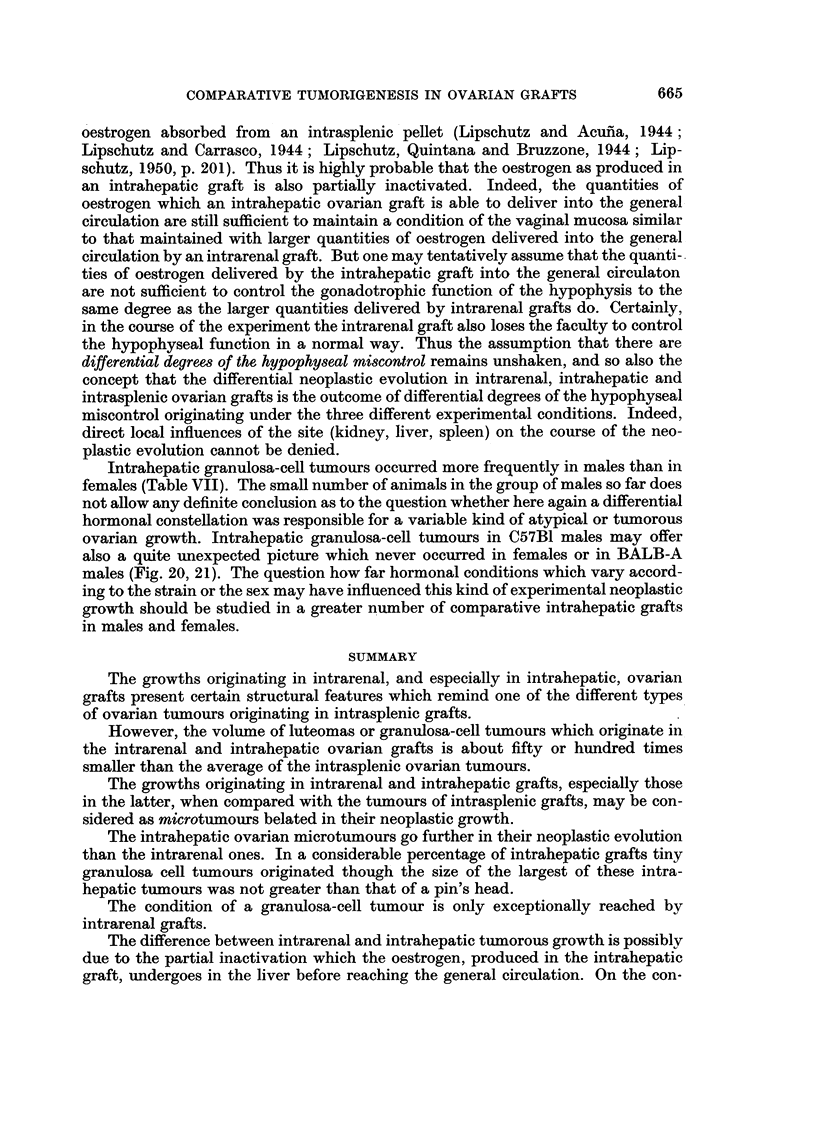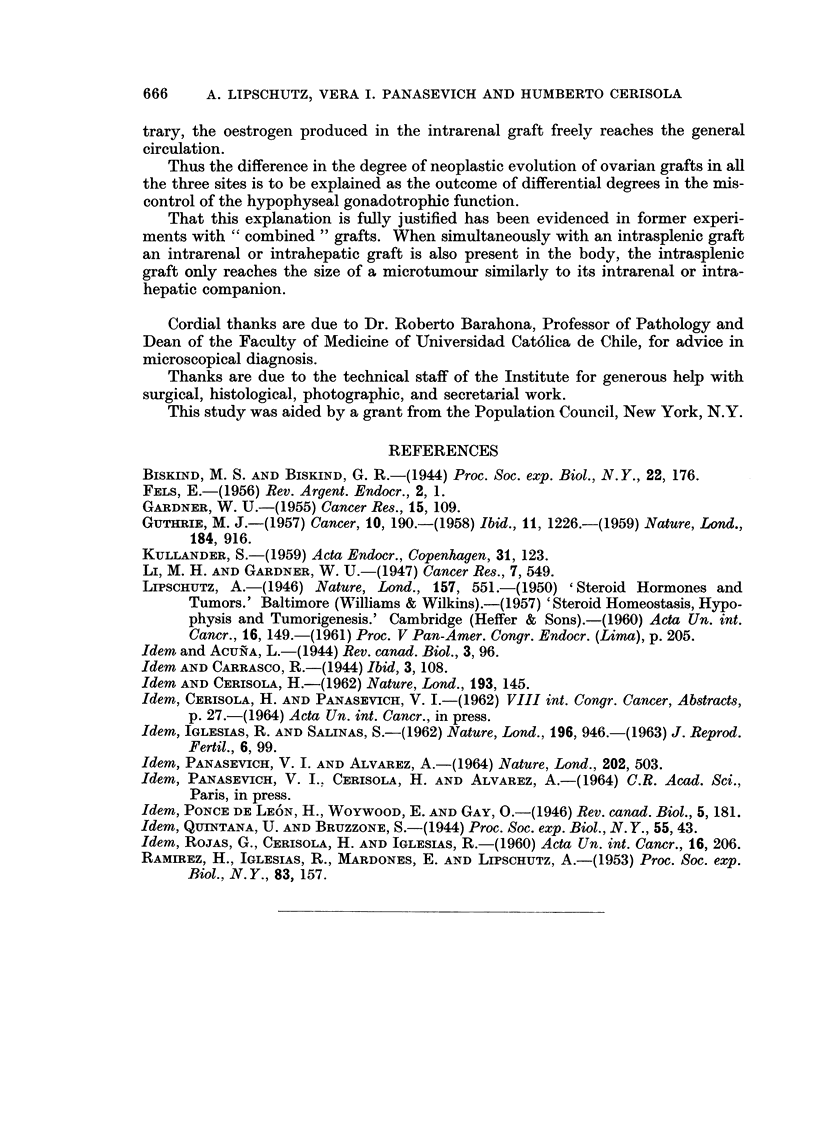# Comparative Tumorigenesis in Intrasplenic, Intrarenal and Intrahepatic Ovarian Grafts

**DOI:** 10.1038/bjc.1964.75

**Published:** 1964-12

**Authors:** A. Lipschutz, Vera I. Panasevich, Humberto Cerisola

## Abstract

**Images:**


### COMPARATIVE TUMORIGENESIS IN INTRASPLENIC, INTRARENAL AND INTRAHEPATIC OVARIAN GRAFTS

A. LIPSCHUTZ, VERA I. PANASEVICH AND HUMBERTO CERISOLA

*From Instituto de Medicina Experimental, Servicio Nacional de Salud, Avenida Irarrázaval 849, Santiago de Chile.*

Received for publication July 29, 1964

THE problem of the comparative behaviour of ovarian grafts at different sites of the body has acquired considerable interest. Originally the idea prevailed that ovarian tumours growing in intrasplenic grafts in the rat were due to an abnormal flow of hypophyseal gonadotrophins, the ovarian hormones produced in the intrasplenic graft being inactivated (or partly inactivated) in the liver before reaching the general circulation, and the normal control of the gonadotrophic activity of the hypophysis failing under the given experimental conditions (Biskind and Biskind, 1944). Later on evidence was produced, that in the rat, tumours appear also in ovaries grafted at other sites of the body as in the kidney and liver (Fels, 1956). Similar statements were made in mice, and it was assumed that these tumours are growing " in response to what can be presumed to be the normal flow of hypophyseal gonadotropins ", " any modification of the hormonal balance " thus being rendered " unlikely " (Guthrie, 1959).

On the other hand, in our work with guinea-pigs haemorrhagic follicles appeared one to two months after grafting the ovary into the spleen (Lipschutz, 1946 ; Lipschutz, Ponce de León, Woywood and Gay, 1946) ; but haemorrhagic follicles failed to appear in intrarenal grafts. Most remarkably, they also failed to appear in intrasplenic ovaries when the latter were combined with intrarenal grafts (Ramirez, Iglesias, Mardones and Lipschutz, 1953). Thus there could be scarcely any doubt that with intrasplenic ovarian grafts, in any case in experiments lasting about two months, the hypophyseal gonadotrophic activity was miscontrolled, contrary to what takes place in experiments with intrarenal ovarian grafts of the same duration. Thus it was only natural that we were not prepared to drop the original concept of hypophyseal imbalance being responsible for ovarian tumorigenesis and to join in the opinion that the appearance of tumours in grafted ovaries was but the outcome of abnormal local conditions. We produced evidence that in mice, in experiments lasting one year or more, tumorigenesis in intrarenal and intrahepatic ovarian grafts was quite different from that in intrasplenic grafts (Lipschutz, 1961). Atypical growth in intrarenal ovarian grafts, even in those cases in which one might be inclined to consider it as tumorous, leads but to microtumours as compared to macrotumours in the spleen ; it is the same with intrahepatic ovarian grafts (Fig. 1). The sequence of the evolutional tumorigenic phases is seemingly the same in intrasplenic, intrahepatic and intrarenal grafts ; but the latter are, both structurally and in size, belated in their evolution.

One may again argue that the striking differences between intrarenal or intrahepatic microtumours, on the one hand, and intrasplenic macrotumours on the other, are due to the different local factors prevailing in the various sites of transplantation. This fundamental problem has been studied in what we called *combined* grafts, one ovary being grafted into the kidney, or liver, the other one into the spleen of the same animal. Under these experimental conditions the intrasplenic growth reaches, similarly to that in the kidney or liver, only the condition of a microtumour, not surpassing, or only slightly surpassing that of intrarenal or intrahepatic growths (Lipschutz, 1961 ; Lipschutz and Cerisola, 1962 ; Lipschutz, Cerisola and Panasevich, 1962, 1964). Thus it became fully evident that the presence of an ovarian graft in the kidney or liver creates in the body a general, or extraovarian, condition which allows only for the growth of ovarian microtumours. And it was but reasonable to suppose that this extraovarian condition is represented by the hypophyseal gonadotrophic function ; and that the miscontrol of the production of hypophyseal gonadotrophins reaches differential degrees according to the site of the graft, or according to the possibility afforded to the ovarian hormones of reaching the general circulation.

The impressive results obtained with combined grafts are not due to competition for hypophyseal gonadotrophins between the two grafts simultaneously present in the body. When both ovaries were grafted into the spleen (double intrasplenic grafts) macrotumours were present in most of the animals ; in many cases there were even two macrotumours in the same spleen (Lipschutz, Panasevich and Alvarez, 1964).

Thus one must assume that the very variable structural condition of ovarian tumours which originate in the body of women is related to the differential degrees of miscontrol of the hypophyseal gonadotrophic function. This might be true even in those cases in which there is, supposedly, some primary failure of the ovary similar to that known experimentally ("subtotal castration" or "ovarian fragmentation"; Lipschutz, 1957, p. 4; 1960).

The problem of the implication of hypophyseal imbalance in ovarian tumorigenesis is of general interest for cancer research. Our communications hitherto have been only very cursory ; in the present paper we shall give a full description of ovarian grafts at different sites of the body ; new knowledge of the comparative condition of these grafts was the starting point for a new study of the problem of hypophyseal imbalance in tumorigenesis (Lipschutz, 1960, 1961 ; Lipschutz and Cerisola, 1962; Lipschutz, Panasevich and Alvarez, 1964, Lipschutz, Panasevich, Cerisola and Alvarez, 1964).

### EXPERIMENTS AND CLASSIFICATION OF RESULTS

Observations on a total of 178 females of two strains (BALB-A and C57Bl) with successful ovarian grafts into the spleen, kidney and liver, are summarized in the present paper. Of these 178 animals, 92 had successful intrasplenic grafts, 38 successful intrarenal and 48 successful intrahepatic grafts ; the duration of the experiments with intrasplenic grafts was 12 months, with intrarenal grafts 12 to more than 15 months, and with intrahepatic grafts 10 and a half to 14 months. Observations on 19 males with intrahepatic grafts have also been included, the total number of animals considered in our comparative analysis thus rising to 197. The animals were operated on by Dr. Elvira Mardones, Dr. Humberto Cerisola and Miss Alicia Alvarez.

The size of the tumours is defined by an index in square millimetres, the surface of the largest section of the tumour in microscope slides. With smaller tumours serial sections of the entire, or almost entire tumour were made. The largest section was designed at ×5. Only tumorous tissue has been considered, omitting as far as possible follicles, cysts or haemorrhagic areas. Most of the determinations were made by Dr. Panasevich. The determination of the index is indeed a very rough method; but the errors occurring with intrasplenic grafts are insignificant in view of the great size of the tumours. Errors are considerable with intrarenal and intrahepatic grafts; but the errors lose any importance when these microtumours are compared with intrasplenic macrotumours. Much more exact is the determination of the index by taking photographs of intrarenal and intrahepatic grafts at a magnification of 35 or 50 and cutting out the tumorous part of the photograph to be weighed. This procedure was applied in 21 cases; the results are summarized in Table III; for the index as found in individual cases, see explanation of Fig. 1, 3, 13, 15, 18, 19, 20, 22–24.

Various authorities have stated that *large* tumours can be found in intrarenal and intrahepatic ovarian grafts. But the large size of the growth is due to cysts which are very frequent in these grafts, and not to tumorous tissue.

The following structural types of atypical growth have been considered in our comparative work: *nodules of " lutein " cells; luteoma* (L; fig. 7C, 17); *luteoma mixed* with granulosa tumour cells, but luteomatous cells prevailing (Lm; Fig. 4B, 11A, 13C, D); *granulosa-cell tumour mixed* with luteomatous cells, but granulosa tumour cells prevailing (Gm; Fig. 12B, 15B, 18B, C, D, 23); *granulosa-cell tumour* (G; Fig. 20B, 21, 22); the so-called *ingrowth* of cells of the ovarian stroma into emptied follicles or cysts; *tubular structures; invagination and excrescence of the germinal epithelium.* There is no difficulty in recognizing the last mentioned structures or in classifying a granulosa-cell tumour. On the contrary, the decision whether a microtumour has to be classified as Lm or Gm is not an easy task; in several cases the classification of the *mixed* tumours was no more than tentative.

For fuller details of the structure of intrasplenic ovarian tumours and of the structural peculiarities of the evolutional phases of intrasplenic ovarian growth we may refer to earlier papers (Li and Gardner, 1947, Gardner, 1955; Guthrie, 1957; Lipschutz, 1960; Lipschutz, Rojas, Cerisola and Iglesias, 1960).

### INTRASPLENIC GRAFTS

Table I summarizes the results obtained in 92 animals with intrasplenic grafts.

TABLE I.—*Intrasplenic Grafts: Predominance of Macrotumours*

| Strain | Total of animals | Age of graft days | Animals with macrotum* | Animals with microtum** | Maximal index mm.² | Minimal index mm.² | Average index mm.² |
|---|---|---|---|---|---|---|---|
| BALB-A | 50 | 363–376† | 43 (86%) | 7 (14%) | 124 | 0·5 | 33±3·6 |
| C57Bl | 42 | 330–377† | 38 (90%) | 4 (10%) | 165 | 0·9 | 33±5·7 |

* With an index not less than 5 mm.²

** With an index less than 5 and not less than 0·5 mm.² For a more critical notion of microtumours see the sections *Intrarenal Grafts* and *Intrahepatic Grafts* in the present paper.

† One animal 307 and 305 days only.

The coincidence of the average index in both large groups is remarkable. But when comparing smaller groups which compose those two given in Table I the average index varies indeed considerably : 26 and 43 mm.$^2$ in BALB-A ; 11·4 (a group of only 9 animals), 32 and 44 mm.$^2$ in C57Bl.

Table II summarizes the structural condition in the 92 animals.

TABLE II.—*Intrasplenic Grafts : Predominance of L in Smaller Tumours*

| Strain | Total of animals | Animals with L and Lm: No. of animals | Animals with L and Lm: Average index mm.$^2$ | Animals with Gm and G: No. of animals | Animals with Gm and G: Average index mm.$^2$ |
|---|---|---|---|---|---|
| BALB-A | 50 | 15 | 16* | 35 | 41 |
| C57Bl | 42 | 8 | 6** | 34 | 39 |

* Eleven animals not reaching the average of 16 mm.$^2$
** Four animals not reaching the average of 6 mm.$^2$

Table II gives evidence that among smaller tumours L and Lm predominate. Gm and G, the culminating point in the neoplastic evolution of the ovary, prevail among the *larger* tumours. In only one *small* tumour of the BALB-A series was Gm found, and in none of the small tumours of the C57Bl series. *The luteomatous condition is a characteristic feature of belated atypical or neoplastic growth in the ovary.*

### INTRARENAL GRAFTS

Table III summarizes results obtained in 38 animals with intrarenal grafts and 67 animals with intrahepatic grafts.

TABLE III.—*Intrarenal and Intrahepatic Grafts : Only Microtumours*

| Site of graft | Strain | Total of animals | Grafts with macrotum. | Grafts with microtum.* | Largest index mm.$^2$ |
|---|---|---|---|---|---|
| FEMALES : | | | | | |
| Kidney | BALB-A | (a) 21 | 0 | 11 | <2 (Fig. 3) |
| | | (b) 17 | 0 | 8 | |
| Liver | BALB-A | 37 | 0 | 19 | >2 (Fig. 24) |
| Liver | C57Bl | 11 | 0 | 5 | |
| MALES : | | | | | |
| Liver | BALB-A | 13 | 0 | 12 | >3 (Fig. 23) |
| Liver | C57Bl | 6 | 0 | 5 | |

* Not less than 0·5 mm.$^2$, but less than 5 mm.$^2$

When speaking of " microtumours " we refer, in the first place, to the fact that in the respective ovary there is a " small " amount of cords or nodules of large, often vacuolated cells (Fig. 2). Thus our notion of microtumours is at the start an *arbitrary* one : those grafts in which the index of luteal cords or nodules reaches 0·5 mm.$^2$ are spoken of as luteomatous microtumours. Indeed, cords of large " lutein " cells are present also in the ovary of our aged BALB-A or C57Bl females (Lipschutz, 1960). But we never found in normal aged animals structural conditions as in our intrarenal grafts of Fig. 2 or others.

In various cases, especially in intrahepatic grafts, there were also other minute structures reminding one of neoplastic growth in intrasplenic ovarian grafts : first, the so called ingrowth of stroma cells into the empty follicles or cysts, and secondly, nodules which can be classified as minute granulosa cell tumours. We shall come back to these preliminary statements after having dealt, separately, with each of the two intrarenal groups mentioned in Table III.

Details of group (*a*) of Table III are given in Table IV :

TABLE IV.—*Intrarenal Ovarian Grafts*, 21 *Animals BALB-A*, *Group* (a) *of Table III. Grafts in the Cortical Region of the Kidney*

Age of grafts : 459 to 463 days ; 1 animal 361 days only

| | Animals with | | Animals with atypical growth | | |
|---|---|---|---|---|---|
| Total of anim. | Follicles I, II, III | Haemorrh. foll. or cysts, incl. haemorrh. lut. cysts | Ingrowth | Nodules of "lutein" cells | L or Lm† |
| 10 | 8 | 6 | 0 | 10* | |
| 11 | 7 | 9 | 1 (Fig. 5) | — | 11** |

* Three with lutein cells in the wall of a large cyst.

** One with lutein cells in the wall of a large cyst. Three animals Lm (Fig. 2, 3, 4) ; one of these possibly Gm (Fig. 3).

† Index not less than $0 \cdot 5$ mm.$^2$

The ovarian graft was found in the cortical region of the kidney. In most of the 21 animals follicles, including haemorrhagic ones, were present. In no less than 15 animals there were haemorrhagic follicles or cysts which is a proof of the existence of a miscontrolled hypophyseal function. As to follicles, or haemorrhagic follicles and cysts, there was seemingly no difference between animals with nodules or cords of $0 \cdot 5$ mm.$^2$ or more (microtumours), and animals with similar nodules of less than $0 \cdot 5$ mm.$^2$

The microtumours in this series are predominantly luteomas, or L, i.e. the nodules consist of large cells sometimes vacuolated. But in several cases Lm was reached, i.e. there were in these cases also nodules reminding one of those present in granulosa-cell tumours (Fig. 2B, 3B and 4B). In two of these grafts follicles were absent.

As already mentioned cysts of variable size were present in these intrarenal grafts (Fig. 4A). As we know from work with intrasplenic grafts these cysts originate from emptied follicles (Guthrie, 1957), possibly also from tubules of the rete. The ovarian stroma surrounding large follicles or cysts may become the site of atypical cellular proliferation (Fig. 4) sometimes with ingrowth into the cyst (Fig. 5).

We shall deal now with the group (*b*) of intrarenal grafts. The results are summarized in Table V.

Two halves of an ovary were introduced into the kidney. In many animals one of the grafts reached the renal pelvis. In 10 out of the 17 animals in which the grafted ovaries had taken the latter were found both in the cortical or medullary region and in the pelvic region, with a total of 20 grafts ; in 3 animals grafts were found only in the pelvic region ; and in 4 animals only in the cortical (or medullary) region. Thus there was a total of 27 grafts.

The pelvic graft adheres to the surface of the medullary region the ovary thus looking into the renal pelvis (Fig. 6, 7, 8).

TABLE V.—*Intrarenal Ovarian Grafts,* 17 *Animals BALB-A, Group* (b) *of Table III. Grafts in the Cortical, or Cortico-medullary, and Pelvic Region of the Kidney*

Total grafts : 27

Age of the grafts : 365 to 375 days

| Site of the graft | Total of grafts | Grafts with: Follicles I, II, III | Grafts with: Haemorrh. foll. or haem. lut. cysts | Grafts with atypical growth: In-growth | Nodules of lut. cells | L or Lm | Gm | Tu-bules | Invagination or excrescence of germ. epith. |
|---|---|---|---|---|---|---|---|---|---|
| Cortical, or cort.-medull. | 14* | 5 | 7 | 4 | 7 | 3** | 2† | 0 | 0 |
| Pelvic | 13* | 1 | 3 | 1 | 7 | 3 | 0 | 10 | 6 |

* Ten animals with both cortico-medullary and pelvic grafts.
** See Fig. 7, 8, 11.
† or Lm ; see Fig. 12 and Table 7.

Contrary to what we have seen with cortical grafts follicles are almost absent in the pelvic region ; haemorrhagic follicles or cysts are also less frequent. There are also other very significant differences in the atypical growth in grafts in the pelvic region compared with grafts in the cortico-medullar region.

In all pelvic grafts the germinal epithelium remained intact. However, the germinal epithelium undergoes two remarkable changes, invagination (Fig. 6B, 8B) and excrescences (Fig. 7B, 9), which were very pronounced in no less than 6 out of 13 pelvic grafts.

Another remarkable feature of atypical growth which is of especial interest from the point of view of neoplastic growth in the ovary, is the occurrence of tubular structures ; they are very rarely found in cortical or medullary grafts but they are present in the overwhelming majority of pelvic grafts (Fig. 6C, 10). Similar tubular structures are occasionally, though rarely, present also in intrahepatic grafts (Fig. 24, 25). We are unable to decide what the origin of these tubular structures is ; they are most probably of follicular origin.

Besides these tubular structures and the proliferation of the germinal epithelium occurring with so great a frequency in ovarian grafts in the pelvic region, there is still another point of difference : the proliferation of the ovarian stroma cells though occurring also in most of the pelvic grafts, is seemingly less pronounced than in cortical or medullar ovarian grafts ; but here also the condition of luteoma can be reached (Fig. 7C, 11).

From comparative observations of cortico-medullar ovarian grafts, on the one hand, and pelvic ovarian grafts on the other hand, two conclusions are reached : (1) the special features of the atypical or tumorous growth in an intrarenal ovarian graft depend to a certain degree upon the site of this graft ; (2) however this atypical growth, though offering some features of neoplastic growth as known from intrasplenic grafts, never reaches the size of the latter and only quite exceptionally the culminating point of ovarian tumorigenesis, the structure of granulosa cell tumours, is reached (only 2 or 3 among 48 grafts ; see Fig. 3 and 12 and Tables IV and V).

It seems idle to discuss the question whether the various features of atypical growth occurring at the different sites of the kidney are already neoplastic or not. Even should one feel inclined to regard the atypical growth occurring in an intrarenal graft as neoplastic and, as to this, similar to that taking place in intrasplenic grafts, one cannot disregard the fact that the intrarenal growths are always *greatly belated* in their neoplastic evolution. This is evidenced, first by the presence of such normal ovarian structures as follicles and corpora lutea in cortical and medullar grafts as late as 15 months after grafting, and especially, by the minute size of those atypical structures which remind one of tumorous growth. If these structures were to be considered as neoplastic, they would certainly be no more than *microtumours* whose volume represents no more, or even less, than about 1 per cent of the average volume of those tumours which originate in intrasplenic ovarian grafts.

### INTRAHEPATIC GRAFTS

We admitted tentatively that the growth taking place in an intrarenal ovarian graft might be considered as the outcome of a greatly belated neoplastic evolution which in various of its features reminds one of that taking place in the spleen. Such a conclusion becomes more evident when comparing intrahepatic ovarian grafts with intrasplenic ones.

The results are summarized in Table VI :

TABLE VI.—*Intrahepatic Ovarian Grafts : 37 Females BALB-A and 11 Females C57Bl, of Table III*

A total of 19 males have also been added

| Strain | Age of grafts days | Total of animals | Animals with: Foll. | Animals with: Haemorrh. foll.* | Animals with: Ingrowth | Animals with: Nodules of " lutein " cells | Animals with: L or Lm | Animals with: Gm or G** |
|---|---|---|---|---|---|---|---|---|
| *Females :* | | | | | | | | |
| BALB-A | 317–429 | 37 | 11 | 14 | 13 | 18 | 11 | 8 |
| C57Bl | 318–430 | 11 | 0 | 7 | 8 | 4 | 3 | 2 |
| *Males :* | | | | | | | | |
| BALB-A | 317–431 | 13 | 2 | 2 | 5 | 1 | 8 | 4 |
| C57Bl | 430 | 6 | 0 | 5 | 4 | 1 | 1 | 4 |

* Including haemorrhagic luteic cysts, haemorrhagic cysts and " haemorrhagic swamps " (Lipschutz, 1960, pp. 151, 152).
** Several animals with tubular structures (Fig. 24, 25).

There is one striking difference between intrarenal and intrahepatic grafts : neoplastic evolution is more rapid in intrahepatic grafts. Among a total of 67 intrahepatic grafts there were no less than 30 with ingrowths into cysts (Fig. 13–21) whereas there were no more than 6 intrarenal grafts with ingrowths among a total of 48 (Fig. 5 and 8 ; see summary in Table IV, V and VI). Also the volume of the ingrowths was much greater in intrahepatic grafts than in intrarenal ones ; a picture of extensive chaotic growth similar to that of intrahepatic grafts as in Fig. 18 to 21 never occurred in intrarenal grafts.

Events in the immediate surroundings of the wall of follicular cysts of intrahepatic grafts, events in the wall itself or in an ingrowth, may offer remarkable pictures of atypical neoplastic growth (Fig. 20, 21). But it is the ingrowth which is undoubtedly one of the most impressive aspects of the vigorous neoplastic growth taking place in experimental ovarian tumours (Guthrie, 1957 ; Lipschutz, Rojas, Cerisola and Iglesias, 1960).

The ingrowth offers also evidence in favour of the fundamental importance which must be attributed to the proliferation of the cells of the ovarian stroma, or interstitial cells, in the genesis of experimental ovarian tumours in mice (Guthrie, 1957) ; the same seems true for similar tumours in the guinea-pig (Lipschutz, 1957) and in the rat (Kullander, 1959). In similar experimental work " granulosa-cell " tumour is certainly a misnomer ! But this does not preclude that tumours of a similar structure may originate also from another cellular source, the germinative epithelium, as insisted upon by Gardner in ample work with intrasplenic grafts in mice (Li and Gardner, 1947) ; and the same may be true also for ovarian tumours induced in mice by steroids (Lipschutz, Iglesias and Salinas, 1962, 1963).

We have referred above to the greater frequency of ingrowths in intrahepatic grafts compared with intrarenal ones. Another very striking difference is the greater frequency of granulosa-cell tumours in intrahepatic grafts, as summarized in Table VII :

TABLE VII.—*Comparative Frequency of Granulosa Cell Tumours in Intrarenal and Intrahepatic Grafts*

| Site of graft | Sex of animals | Age of the grafts months | Number of grafts | Grafts with Gm and G | | References | |
|---|---|---|---|---|---|---|---|
| | | | | Number | % | Table | Fig. |
| Kidney | fem. | 12–15 | 48 | 3 (or 1*) | 6 (or 2) | 4, 5 | 3, 12 |
| Liver | fem. | 12–14 | 48 | 10** | 21 | 6 | 15, 18, 22, 24, 25 |
| Liver | male | 12–14 | 19 | 8 | 42 | 6 | 20, 21, 23 |

* Two animals possibly Lm.
** One animal with tubular structures predominating (Fig. 25).

The granulosa-cell tumours were mostly of the mixed type, with relatively small or large areas of lutein cells (Fig. 15, 18, 23). Though it was, here again, sometimes difficult to decide whether an intrahepatic microtumour had to be classified as Lm of Gm, these difficulties were less great than with intrarenal grafts.

An intraheptic granulosa-cell tumour with complete, or almost complete absence of large lutein cells, is shown in Fig. 22.

We have already referred to the remarkable picture the ingrowths may present (Fig. 20, 21). In these cases a bizarre structure resulted from the ingrowth, appearing in the section as a complex network (Fig. 20), or partly " arborized " (Fig. 21). There were 3 intrahepatic grafts which presented this picture. All the 3 cases occurred among the 4 C57Bl *males* with granulosa-cell tumours ; there was no similar case among the 4 granulosa-cell tumours in BALB-A males, nor among the 10 granulosa-cell tumours in *females* of both strains.

Another remarkable feature is the presence of small tubular structures in granulosa-cell tumours (Fig. 24) ; tubular structures may even predominate (Fig. 25). They are very similar to the tubules which are present in many grafts in the pelvic region of the kidney (Fig. 6, 10).

In 4 out of the 18 intrahepatic grafts with Gm or G in Table VII follicles (primary, tertiary or haemorrhagic) were present. These four tumours belonged to the mixed type. But follicles also occurred with a similar small frequency in the remaining 49 intrahepatic grafts (15 grafts with follicles). Large haemorrhagic cysts, also, were present in several animals of both comparative groups.

In experiments with combined grafts (an intrarenal, or intrahepatic, graft together with an intrasplenic one in the same animal) the intrarenal and intrahepatic grafts offer a picture identical with that described in this paper for single grafts. There was but one exception : among 72 intrarenal and intrahepatic grafts combined with intrasplenic ones there was *one* case with an intrahepatic macrotumour (a granulosa-cell tumour of considerable size ; for quantitative summary see Lipschutz, Panasevich, Cerisola and Alvarez, 1964). This finding is all the more difficult to explain as the accompanying intrasplenic graft was in this case a microtumour, as is the rule with combined grafts !

### DISCUSSION

Intrahepatic ovarian grafts, like intrarenal ones, remain microtumours even when the experiment is prolonged for as long as 15 months. But it is evident that intrahepatic grafts, though also greatly delayed in their neoplastic evolution when compared with intrasplenic grafts, are structurally nearer to the latter than intrarenal grafts.

As shown above, the evolution of atypical growth or, if one likes, of neoplastic growth in intrarenal grafts is very different in the pelvic region from that in the cortical and medullary regions. At first sight one may then be inclined to explain the pronounced differences between intrarenal and intrahepatic grafts as due to local influences. However, there is also the question whether the hormonal constellation so far as it depends upon the ovary, is similar or different in intrarenal and intrahepatic grafts. Some insight into the latter problem can be obtained by examining the vaginal mucosa. The results are summarized in Table VIII :

TABLE VIII.—*Vaginal Mucosa, at the End of Experiments*

| Site of the graft | Total of animals | Anoestr. | Prooestr. | Oestr. | Metoestr. | References |
|---|---|---|---|---|---|---|
| Intrasplenic | 29 | 6 | 7 | 14 | 2 | Table I |
| Intrarenal. | 34 | 0 | 6 | 26 | 2 | Table IV & V |
| Intrahepatic | 29 | 0 | 7 | 18 | 4 | Table VI |
| | | % | % | % | % | |
| Intrasplenic | 100 | 21 | 24 | 48 | 7 | |
| Intrarenal | 100 | 0 | 18 | 76 | 6 | |
| Intrahepatic | 100 | 0 | 24 | 62 | 14 | |

There is a pronounced difference between the hormonal behaviour of intrasplenic ovarian grafts and intrarenal, or intrahepatic, grafts. Not a single case of anoestrus was found among 63 animals with intrarenal and intrahepatic grafts, whereas no less than 21 per cent of animals with intrasplenic grafts were in anoestrus at the moment of necropsy. On the other hand there was, as to oestrogenic activity, no difference between intrarenal and intrahepatic grafts.

Does the above result mean that the structural differences between intrarenal and intrahepatic grafts cannot be related to a differential hormonal constellation? Experimental evidence has been produced that oestrogen absorbed from an intrahepatic pellet is partly inactivated, though not on the same quantitative scale as

### EXPLANATION OF PLATES

FIG. 1.—×4½.—A. Intrasplenic graft of *average* size, 368 days. C57Bl. Index 33 mm.$^2$ Granulosa-cell tumour mixed.—B. Intrarenal graft of *large* size, 462 days. BALB-A. Index 1·9 mm.$^2$ Luteoma mixed, or granulosa-cell tumour mixed. See Fig. 3.—C. Intrahepatic graft of *large* size, 318 days. C57Bl. Index 1·9 mm.$^2$ Granulosa-cell tumour. See Fig. 22.

FIG. 2.—Intrarenal graft, 361 days. BALB-A. Cortico-medullary region. Tertiary and haemorrhagic follicles present. Classified as Lm.—A. Nodules of lutein cells ×180.—B. Nodules of smaller cells, possibly of tubular (or follicular) origin. ×180.

FIG. 3.—Intrarenal graft, 462 days. BALB-A. No follicles. Classified as Lm, possibly Gm.—A. Graft in the cortico-medullary region. Cysts. Index : 1·9 mm.$^2$ ×32.—B. Nodules of small cells of granulosa-type ; larger cells also present. ×180.

FIG. 4.—Intrarenal graft, 462 days. BALB-A. Cortico-medullary. No follicles. Classified as Lm.—A. The tumour originated in the wall of a large cyst. ×32.—B. Nodules of large and small cells. ×180.

FIG. 5.—Intrarenal graft, 463 days. BALB-A. Cortico-medullary. Tertiary and haemorrhagic follicles present. Ingrowth of large cells into an haemorrhagic cyst. ×180.

FIG. 6.—Intrarenal graft, pelvic, 375 days. BALB-A.—A. Graft looking into the renal pelvis. ×32.—B. Invagination of the germinal epithelium. ×90.—C. Abundant tubular structures. Nodules of large cells. ×90.

FIG. 7.—Intrarenal graft, pelvic, 375 days. BALB-A. Classified as L.—A. Graft looking into the renal pelvis. ×32.—B. Excrescence of germinal epithelium. ×180.—C. Nodules of cells of variable size. ×180.

FIG. 8.—Cortico-medullary and pelvic graft in immediate mutual contact. 365 days. BALB-A. —A. Two large cysts in the cortico-medullary graft ; one cyst with ingrowth. Pelvic graft with invagination of germinal epithelium and excrescences. ×32.—B. Invaginations of germinal epithelium in pelvic graft. ×180.—C. Ingrowth in cortico-medullary graft. Classified as L (?). ×180

FIG. 9.—Intrarenal graft, pelvic, 375 days. BALB-A. Excrescence. Haemorrhagic luteic cyst. Tubular (or follicular) structures. ×90.

FIG. 10.—Intrarenal graft, pelvic, 375 days. BALB-A. Abundant tubular structures. ×180.

FIG. 11.—Intrarenal graft, pelvic, 365 days. Classified as Lm.—A. ×32.—B. ×180.

FIG. 12.—Intrarenal graft, cortico-medullary, 375 days. BALB-A. Classified as Gm, possibly Lm.—A. ×32.—B. ×180.

FIG. 13.—Intrahepatic graft, 365 days. BALB-A. Classified as Lm. Index : 0·9 mm.$^2$—A. Part of the graft showing two ingrowths. ×32.—B. The ingrowths of the left side. ×180.—C. Ingrowth of the right side. ×180.—D. Large nodules of lutein and granulosa-type cells. ×180.

FIG. 14.—Intrahepatic graft, 365 days. BALB-A.—A. Large ingrowth. ×32.—B. Mostly lutein cells. ×180.

FIG. 15.—Intrahepatic graft, 364 days. BALB-A. Classfied as Gm. Index : 0·8 mm.$^2$—A. ×32.—B. Nodules of granulosa-type cells and of lutein cells. ×180.

FIG. 16.—Intrahepatic graft, 364 days. BALB-A. Several ingrowths. Granulosa-type cells. ×180.

FIG. 17.—Intrahepatic graft, 429 days. BALB-A. Classified as L. Chaotic ingrowths. ×90.

FIG. 18.—Intrahepatic graft, 424 days. BALB-A. Classified as Gm. Index : 1·5 mm.$^2$—A. Ingrowths. ×32.—B. ×90.—C. Top—area of granulosa-type cells ; bottom—area of lutein cells. ×180.—D. Nodules of both types of cells intermingling. ×180.

FIG. 19.—Intrahepatic graft, 320 days. BALB-A, *male*. Classified as Lm. Index : 0·8 mm.$^2$ Multiple ingrowths. ×90.

FIG. 20.—Intrahepatic graft, 430 days. C57Bl, *male*. Classified as G. Index : 2·2 mm.$^2$—A. Bizarre structure resulting from an ingrowth. ×32.—B. ×90.

FIG. 21.—Intrahepatic graft, 430 days. C57Bl, *male*. Classified as G. ×90.

FIG. 22.—Intrahepatic graft, 318 days. C57Bl. Classified as G. Index : 1·9 mm.$^2$—A. ×32.—B. ×180.

FIG. 23.—Intrahepatic graft, 431 days. BALB-A, *male*. Classified as Gm. Index : 3·3 mm.$^2$ —A. On the right, nodules of lutein cells ; on the left, part of the granulosa-cell tumour. ×32.—B. Granulosa-cell tumour. ×180.

FIG. 24.—Intrahepatic graft, 427 days. BALB-A. Classified as Gm. Index : 2·1 mm.$^2$—A. To the left, lutein and granulosa-type cells ; to the right, tubular structures. ×32.—B. Tubular part. ×180.

FIG. 25.—Intrahepatic graft, 365 days. C57Bl. Abundance of tubular structures. Classified as Gm. Tubules and nodules of lutein cells and of cells of a smaller size. ×90.

Lipschutz, Panasevich and Cerisloa.

4A

4B

5

6A

6B

6C

Lipschutz, Panasevich and Cerisola.

7A

7B

9

7C

8A

Lipschutz, Panasevich and Cerisola.

8C

8B

10

11A

11B

Lipschutz, Panasevich and Cerisola.

12A

12B

13A

13C.

13B

13D

14A

14B

15A

15B

17

16

Lipschutz, Panasevich and Cerisola.

18A

18B

18C

18D

Lipschutz, Panasevich and Cerisola.

20A

21

20B

19

25

Lipschutz, Panasevich and Cerisola.

22A

22B

23A

23B

24A

24B

Lipschutz, Panasevich and Cerisola.

oestrogen absorbed from an intrasplenic pellet (Lipschutz and Acuña, 1944; Lipschutz and Carrasco, 1944; Lipschutz, Quintana and Bruzzone, 1944; Lipschutz, 1950, p. 201). Thus it is highly probable that the oestrogen as produced in an intrahepatic graft is also partially inactivated. Indeed, the quantities of oestrogen which an intrahepatic ovarian graft is able to deliver into the general circulation are still sufficient to maintain a condition of the vaginal mucosa similar to that maintained with larger quantities of oestrogen delivered into the general circulation by an intrarenal graft. But one may tentatively assume that the quantities of oestrogen delivered by the intrahepatic graft into the general circulaton are not sufficient to control the gonadotrophic function of the hypophysis to the same degree as the larger quantities delivered by intrarenal grafts do. Certainly, in the course of the experiment the intrarenal graft also loses the faculty to control the hypophyseal function in a normal way. Thus the assumption that there are *differential degrees of the hypophyseal miscontrol* remains unshaken, and so also the concept that the differential neoplastic evolution in intrarenal, intrahepatic and intrasplenic ovarian grafts is the outcome of differential degrees of the hypophyseal miscontrol originating under the three different experimental conditions. Indeed, direct local influences of the site (kidney, liver, spleen) on the course of the neoplastic evolution cannot be denied.

Intrahepatic granulosa-cell tumours occurred more frequently in males than in females (Table VII). The small number of animals in the group of males so far does not allow any definite conclusion as to the question whether here again a differential hormonal constellation was responsible for a variable kind of atypical or tumorous ovarian growth. Intrahepatic granulosa-cell tumours in C57Bl males may offer also a quite unexpected picture which never occurred in females or in BALB-A males (Fig. 20, 21). The question how far hormonal conditions which vary according to the strain or the sex may have influenced this kind of experimental neoplastic growth should be studied in a greater number of comparative intrahepatic grafts in males and females.

### SUMMARY

The growths originating in intrarenal, and especially in intrahepatic, ovarian grafts present certain structural features which remind one of the different types of ovarian tumours originating in intrasplenic grafts.

However, the volume of luteomas or granulosa-cell tumours which originate in the intrarenal and intrahepatic ovarian grafts is about fifty or hundred times smaller than the average of the intrasplenic ovarian tumours.

The growths originating in intrarenal and intrahepatic grafts, especially those in the latter, when compared with the tumours of intrasplenic grafts, may be considered as *micro*tumours belated in their neoplastic growth.

The intrahepatic ovarian microtumours go further in their neoplastic evolution than the intrarenal ones. In a considerable percentage of intrahepatic grafts tiny granulosa cell tumours originated though the size of the largest of these intrahepatic tumours was not greater than that of a pin's head.

The condition of a granulosa-cell tumour is only exceptionally reached by intrarenal grafts.

The difference between intrarenal and intrahepatic tumorous growth is possibly due to the partial inactivation which the oestrogen, produced in the intrahepatic graft, undergoes in the liver before reaching the general circulation. On the contrary, the oestrogen produced in the intrarenal graft freely reaches the general circulation.

Thus the difference in the degree of neoplastic evolution of ovarian grafts in all the three sites is to be explained as the outcome of differential degrees in the mis-control of the hypophyseal gonadotrophic function.

That this explanation is fully justified has been evidenced in former experiments with " combined " grafts. When simultaneously with an intrasplenic graft an intrarenal or intrahepatic graft is also present in the body, the intrasplenic graft only reaches the size of a microtumour similarly to its intrarenal or intrahepatic companion.

Cordial thanks are due to Dr. Roberto Barahona, Professor of Pathology and Dean of the Faculty of Medicine of Universidad Católica de Chile, for advice in microscopical diagnosis.

Thanks are due to the technical staff of the Institute for generous help with surgical, histological, photographic, and secretarial work.

This study was aided by a grant from the Population Council, New York, N.Y.

### REFERENCES

BISKIND, M. S. AND BISKIND, G. R.—(1944) *Proc. Soc. exp. Biol., N.Y.*, **22**, 176.
FELS, E.—(1956) *Rev. Argent. Endocr.*, **2**, 1.
GARDNER, W. U.—(1955) *Cancer Res.*, **15**, 109.
GUTHRIE, M. J.—(1957) *Cancer*, **10**, 190.—(1958) *Ibid.*, **11**, 1226.—(1959) *Nature, Lond.*, **184**, 916.
KULLANDER, S.—(1959) *Acta Endocr., Copenhagen*, **31**, 123.
LI, M. H. AND GARDNER, W. U.—(1947) *Cancer Res.*, **7**, 549.
LIPSCHUTZ, A.—(1946) *Nature, Lond.*, **157**, 551.—(1950) 'Steroid Hormones and Tumors.' Baltimore (Williams & Wilkins).—(1957) 'Steroid Homeostasis, Hypophysis and Tumorigenesis.' Cambridge (Heffer & Sons).—(1960) *Acta Un. int. Cancr.*, **16**, 149.—(1961) *Proc. V Pan-Amer. Congr. Endocr. (Lima)*, p. 205.
*Idem* and ACUÑA, L.—(1944) *Rev. canad. Biol.*, **3**, 96.
*Idem* AND CARRASCO, R.—(1944) *Ibid*, **3**, 108.
*Idem* AND CERISOLA, H.—(1962) *Nature, Lond.*, **193**, 145.
*Idem*, CERISOLA, H. AND PANASEVICH, V. I.—(1962) *VIII int. Congr. Cancer, Abstracts*, p. 27.—(1964) *Acta Un. int. Cancr.*, in press.
*Idem*, IGLESIAS, R. AND SALINAS, S.—(1962) *Nature, Lond.*, **196**, 946.—(1963) *J. Reprod. Fertil.*, **6**, 99.
*Idem*, PANASEVICH, V. I. AND ALVAREZ, A.—(1964) *Nature, Lond.*, **202**, 503.
*Idem*, PANASEVICH, V. I., CERISOLA, H. AND ALVAREZ, A.—(1964) *C.R. Acad. Sci.*, Paris, in press.
*Idem*, PONCE DE LEÓN, H., WOYWOOD, E. AND GAY, O.—(1946) *Rev. canad. Biol.*, **5**, 181.
*Idem*, QUINTANA, U. AND BRUZZONE, S.—(1944) *Proc. Soc. exp. Biol., N.Y.*, **55**, 43.
*Idem*, ROJAS, G., CERISOLA, H. AND IGLESIAS, R.—(1960) *Acta Un. int. Cancr.*, **16**, 206.
RAMIREZ, H., IGLESIAS, R., MARDONES, E. AND LIPSCHUTZ, A.—(1953) *Proc. Soc. exp. Biol., N.Y.*, **83**, 157.